# A Statistical Physics Characterization of the Complex Systems Dynamics: Quantifying Complexity from Spatio-Temporal Interactions

**DOI:** 10.1038/srep27602

**Published:** 2016-06-14

**Authors:** Hana Koorehdavoudi, Paul Bogdan

**Affiliations:** 1 Department of Aerospace and Mechanical Engineering, University of Southern California, Los Angeles, CA 90089-1453, USA; 2 Department of Electrical Engineering, University of Southern California, Los Angeles, CA 90089-2560, USA

## Abstract

Biological systems are frequently categorized as complex systems due to their capabilities of generating spatio-temporal structures from apparent random decisions. In spite of research on analyzing biological systems, we lack a quantifiable framework for measuring their complexity. To fill this gap, in this paper, we develop a new paradigm to study a collective group of *N* agents moving and interacting in a three-dimensional space. Our paradigm helps to identify the spatio-temporal states of the motion of the group and their associated transition probabilities. This framework enables the estimation of the free energy landscape corresponding to the identified states. Based on the energy landscape, we quantify missing information, emergence, self-organization and complexity for a collective motion. We show that the collective motion of the group of agents evolves to reach the most probable state with relatively lowest energy level and lowest missing information compared to other possible states. Our analysis demonstrates that the natural group of animals exhibit a higher degree of emergence, self-organization and complexity over time. Consequently, this algorithm can be integrated into new frameworks to engineer collective motions to achieve certain degrees of emergence, self-organization and complexity.

A complex system refers to a system in which there is a lack of precise relation between the system’s outcomes and the original causes of those outcomes^1^,^2^,^3^,^4^. The main characteristics of complex systems are their unpredictable and nonlinear dynamics. This complexity in a system is due to the intricate heterogeneous coupling between the components of the system, which makes it impossible to analyze the components individually and isolated from the rest of the system^5^. The close coupling and interactions between the units of the complex system cause recognizable collective behavior at larger scales^6^.

A group of agents or animals moving collectively is an example of a complex system. The collective characteristics between the agents of a group are regulated by behavioral tendencies, as well as short-range and long-range interactions among them. In such a complex system, the group with identical agents’ behavior evolves through different states (i.e., spatio-temporal arrangement/configuration of the agents moving in a collective group formation)^7^,^8^. We can encode the dynamics (evolution) of the group among different states by constructing a free energy landscape representation^9^,^10^,^11^,^12^,^13^,^14^,^15^,^16^,^17^,^18^,^19^,^20^,^21^. Different factors like the number of members, internal capabilities of the individuals (e.g., sensitivity to neighbors, motion speed of individuals, computational/processing capabilities of agents) and external properties (e.g., environmental and boundary condition) influence the overall collective behavior and the free energy landscape; further, these factors can contribute to various phase transitions in the group structure among several possible states in the corresponding free energy landscape.

Despite significant research and progress in studying natural^22^,^23^,^24^,^25^,^26^,^27^,^28^,^29^,^30^ and engineered^31^,^32^,^33^,^34^,^35^,^36^,^37^,^38^,^39^,^40^,^41^,^42^,^43^ collective systems, the field is still trying to quantify the dynamical states in a collective motion and predict the transition between them. Toward this end, in this paper, we develop a new approach, which for the first time identifies and extracts the dynamical states of the spatial formation and structure for a collective group. Our mathematical framework enables the estimation of the free energy landscape of the states of the group motion and also quantifies the transitions among them. In this approach, we are able to distinguish between stable and transition states in a motion by differentiating them according to their energy level and the amount of time the group prefers to stay in each state. We noticed the collective group has a lower energy level at stable states compared to transition ones. This could be the reason for which the group prefers to stay for a relatively longer time in stable states compared to transition states during their motion. Furthermore, the group’s structure may convert to one of the possible transition states with higher energy level while reorganizing itself and evolving between two different stable states with different spatial organization.

To provide a quantifiable approach for the collective motion complexity, based on the newly described free energy landscape, we introduce first the concept of missing information related to spatio-temporal conformation of a group motion and then quantify the emergence, self-organization and complexity associated with the exhibited spatial and temporal group dynamics. We define these metrics for a collective motion based on general definitions in information theory presented by Shannon^44^,^45^. Our approach enables a mathematical quantification of biological collective motion complexity. Furthermore, this framework allows us to recognize and differentiate among various possible states based on their relative energy level and complexity measures. Identifying these dynamical states opens the avenue in robotics for developing engineered collective motions with desired level of emergence, self-organization and complexity. For example, if a particular set of states and their dependent structure correspond to a highly robust yet agile collective motion, then one can use this information theoretic inspired metrics for engineering the agent-to-agent interactions rather than focusing on the highly expensive computation strategy for an agent based model to achieve a certain degree of emergence, self-organization and complexity. We clarify this further in discussion section of manuscript. This framework can also help to study the evolution of the motion of various animal groups in nature to better understand their means to achieve energy efficiency^46^.

The remaining of this paper is organized as follows: In the first section of results, we present our framework to extract the possible states in the collective motion and the strategy to build the corresponding energy landscape for transitions between them. To demonstrate the benefits of our approach, we first apply this strategy to quantify the energy landscape of a self-organizing model of a simulated group of agents based on local interactions among its individuals. Next, we define the missing information for the group structure. In the second section, we apply the same framework to three natural groups of swimming bacteria, flying pigeons and ants and study their energy landscapes. We define emergence, self-organization, and quantify the complexity of a collective motion based on these newly introduced metrics. For the case of bacteria, we concluded that adding chemoattractant to the environment, decreases the number of possible states for the group motion and the free energy landscape is smoother compared to the case without chemoattractant. Finally, the discussion section concludes the paper and outlines some future research directions.

## Results

### Estimating the free energy landscape for a collective motion based on identified spatio-temporal structural states of the group

**Figure 1:**
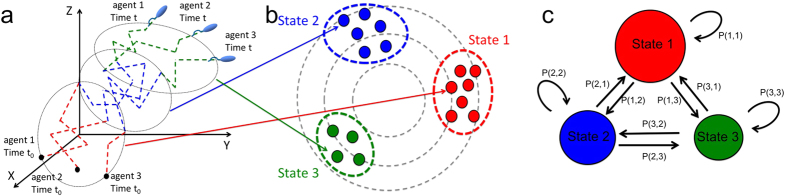
Schematic description of the main steps for building the energy landscape for a group of N agents moving in a three-dimensional space. (**a**) First, we subdivide the trajectories of all agents in the group to equal sub-intervals centered at time ***t***_**c**_ with a time window of [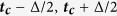], where Δ is the predefined time scale. Next, we estimate the three-dimensional probability distribution function of the motion of the group for each sub-interval. (**b**) We use the Kantorovich metric to cluster these sub-interval time series based on their similarities in the probability distribution function. Each cluster of sub-intervals can be interpreted as a state for the collective motion. (**c**) In the last step, we estimate the transition probability matrix among the identified states of the collective motion.

The agents move coherently within a collective group while interacting with their immediate neighbors and determine their overall trajectory of motion with respect to other agents. Consequently, the group’s structure evolves among various spatio-temporal structural states. We can identify and extract these states of the group moving in three-dimensional space from the individuals’ trajectories using our algorithm explained as follow (see the free energy landscape section in the Methods for more details). First, we divide the trajectories of all the individuals into equal sub-intervals of a specific lenght. Next, we compute the multivariable probability distribution function of the location of all the individuals in every sub-interval (Fig. 1a). We use Kantorovich metric (see equation (5) in free energy landscape section in Methods) to cluster these subinterval time series based on their similarities and closeness in the probability distribution function (Fig. 1b). Each cluster contains subintervals with similar dynamical configuration and can be interpreted as a distinct state. In the next step, we estimate the transition probability matrix between the identified states (Fig. 1c) and based on these probabilities, we are able to construct the free energy landscape for the transitions between these states (see equations (6) and (7) in free energy landscape section in Methods). Our formalism generalizes the method of local equilibrium state analysis presented by Akinori Baba and his coworkers^13^,^47^ to construct the energy landscape of a one-dimensional single molecule time series. To further exploit this free energy landscape description, we develop an information theoretic framework for quantifying the degree of emergence, self-organization and complexity of a collective group motion.

To analyze our framework, we first use a well-known agent-based model^31^ which captures different behavior of a collection of interactive agents in a three dimensional space (see the simulation section in the Methods for more details about the model). This model is based on simplified local interactions between the individuals and is able to mimic the spatial dynamics of a group of animals such as bird flocks or fish school. By varying the degree of local interactions among the agents in this model, we can observe different types of behavior from the group^31^. The motion of each individual in the group is the outcome of local repulsion, alignment and attraction tendencies depending on the location and orientation of the neighboring agents. The individuals tend to align themselves with the neighbors, while avoiding collision by keeping a minimum distance between them. Individuals avoid being isolated and keep the group to move as a single coherent entity by maintaining an attraction tendency between them.

**Figure 2:**
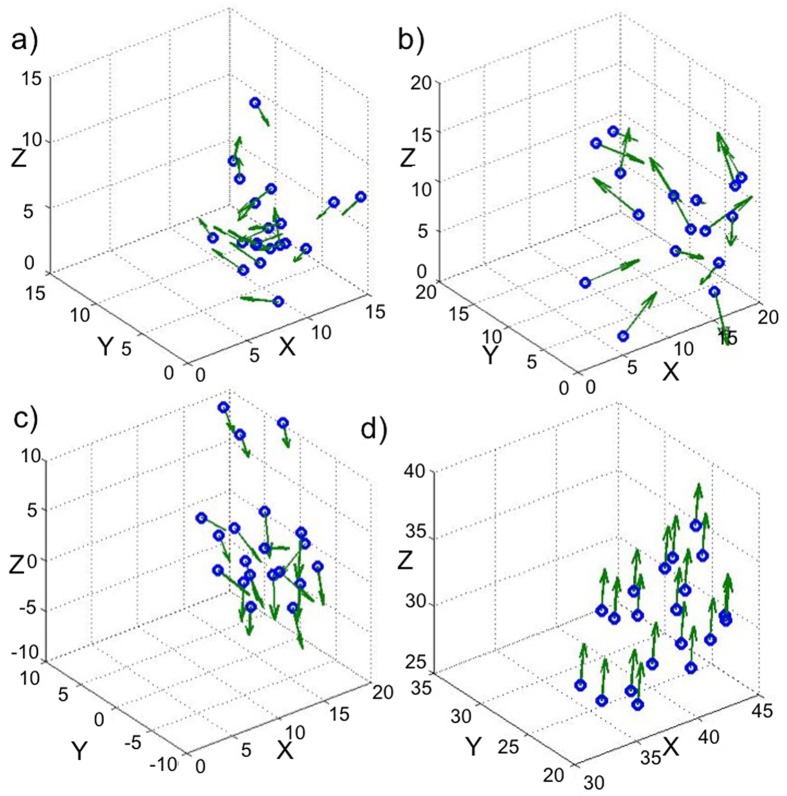
Various collective patterns of a simulated model of a group of agents moving in a three dimensional space. (**a**) *Torus:* Individuals rotate around a center point within an empty space (See the simulation section in the Methods for more details about the model). (**b**) *Swarm:* Individuals show attraction and repulsion behavior between themselves and there is no orientation behavior consequently no parallel motion. (**c**) *Dynamic parallel group:* Individuals align with each other and make the group more motile compared to two previous cases. (**d**) *Highly parallel group:* Individuals are in a highly aligned arrangement and the group is more motile compared to previous cases.

The dynamics of the group can change between four different common collective behaviors depending on the width of different zones around the individuals (Fig. 2). These four collective motion behaviors identified by Couzin and coworkers^31^ are: *torus*, *swarm, dynamic parallel group* and *highly parallel group*. The *torus* configuration emerges when the individuals rotate around an empty space. This happens when the *zone of orientation* is relatively small compared to *zone of attraction*. In this case individuals have a tiny *zone of repulsion* around them (Fig. 2a). On the other hand, when individuals exhibit an attraction or repulsion behavior between themselves and there is no orientation behavior (consequently no parallel motion), the colony demonstrates a so-called *swarm* collective behavior (Fig. 2b). The *dynamic parallel group* emerges by increasing the *zone of orientation*, which causes the individuals align with each other and makes the group more motile compared to the above-mentioned two cases (Fig. 2c). The group shifts to a *highly parallel group behavior* when the *zone of orientation* is relatively bigger compared to the case of *dynamic parallel group* (Fig. 2d). In this case, the individuals are in a highly aligned arrangement and the group is more motile compared to the previous cases^31^.

**Figure 3:**
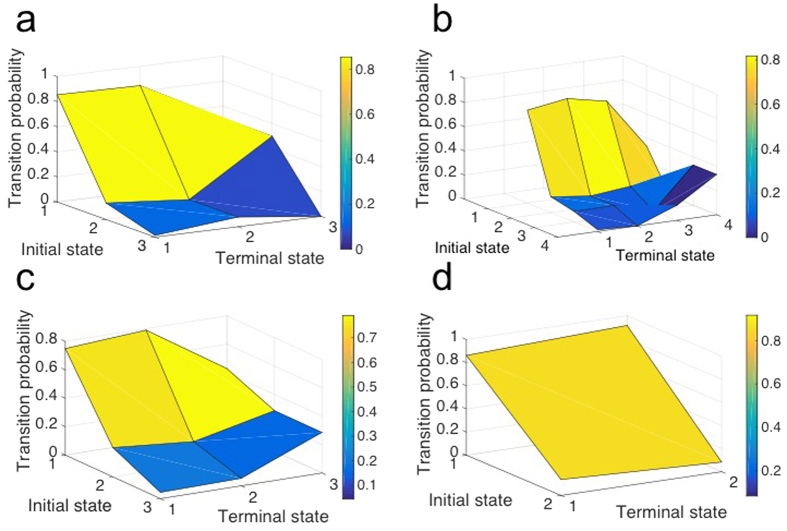
Transition probabilities among the states identified in different collective behaviors of the simulated agent-based model. (**a**) *Torus*, the plot shows the transition probability between different states in this collective behavior. (**b**) *Swarm*, the group of agents has the highest number of states in this collective behavior and the landscape has more spikes and is less smooth compared to the other cases. (**c**) *Dynamic parallel group*, the transition probability looks similar to the *torus* phase, this similarity is due to preference of individuals to align their motion parallel to their neighbors. (**d**) *Highly parallel group*, the group has the lowest number of possible states in this phase and the landscape is less spiky due to high preference of individuals to move parallel with respect to each other.

We analyze these four types of collective behavior separately using our free energy landscape framework and show that: (1) each behavior is a combination of various structural states and (2) the group is transitioning among these spatio-temporal states over time. We identify and extract these states as building blocks for each collective behavior type. Figure 3 summarizes the transition probabilities between them for each case. We use the estimated transition probabilities to compute the free energy landscape using equation (6) and (7) in the free energy landscape section in Methods. Comparison between different cases shows that *swarm* behavior evolves between more possible states and this confirms that there is lower level of arrangement in the group in *swarm* phase. As a result, the transition matrix and correspondingly its energy landscape for this case has more spikes and is less smooth compared to the other cases (Fig. 3b). In contrast, for *torus* and *dynamic parallel group* behaviors there is more structural order due to alignment and parallel motion between individuals. Consequently, the collective dynamics of the group is characterized by fewer possible states when compared to *swarm* behavior and the transition matrix and energy landscape is smoother (Fig. 3a-3c). For *highly parallel group* all the individuals are completely aligned with each other and the structure of the group has the highest arrangement compared to the other behaviors, so the structure of the group evolves between the lowest number of possible structural states and the transition probability matrix is less spiky (Fig. 3d).

### Missing information of a group’s structure can be quantified using the transition probability matrix among the identified structural states

**Figure 4:**
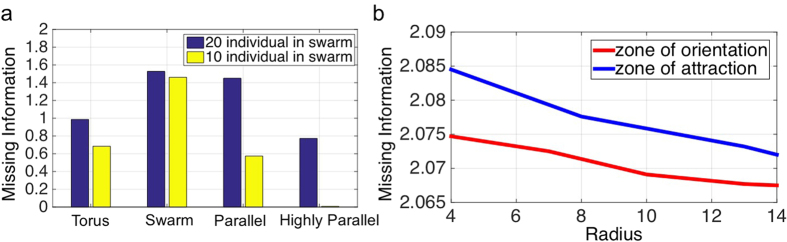
Quantifying the missing information of the entire simulated agent-based model for various interaction rules. (**a**) We quantify the missing information from the dynamics of a group of agents considering different interaction rules which causes various collective behavior in the group while considering the same initial condition for the agents position. This plot shows the transition from *swarm* phase to *torus*, *dynamic parallel group* and then *highly parallel group* and the fact that the missing information is decreasing due to an increase in the internal order of the group. (**b**) The quantified missing information extracted from the group dynamics when the population consists of 100 individuals for varying value of the radius of *zone of orientation* while radii of *zone of repulsion* and *attraction* is fixed (red line) and in other case varying value of the radius of *zone of attraction* while radii of *zone of repulsion* and *orientation* is fixed (blue line).

In what follows, we quantify the missing information of the group for different types of collective behavior related to the model presented in the previous section. The missing information has been defined as a way to quantify the number of possible different ways to arrange a specific system^44^,^48^. Therefore, it estimates the level of disorder in a system. For a collective motion, we consider it as a level of missing communicated information between the agents due to their interactions with each other. This can be interpreted as the amount of information needed to specify the exact physical state of the group forming a specific spatial arrangement. This also means how much internal order or uncertainty a group formation has in a specific state. Figure 4a compares the missing information of the swarm for different collective behaviors with different number of individuals (see missing information section in Methods). In our analysis, we consider the same initial conditions for the individuals’ location and speed. The radii of individuals’ interaction zones generating similar collective behavior are identical irrespective of the population size of the group. Figure 4a shows the transition from a *swarm* phase to *torus*, *dynamic parallel group* and then *highly parallel group;* corresponding to this transition, we observe that the missing information is decreasing due to more alignment among the neighbors and increasing internal order of the group structure.

To further investigate the effect of local interactions on missing information related to the group structure, we increase the group density to 100 individuals, we fix the radius of *zone of repulsion* and *zone of orientation,* and we change the *zone of attraction.* We consider the same initial condition for the individuals’ location and speed in all the cases. We perform the same analysis and quantify the missing information of the collective group. Figure 4b (blue line) shows that by increasing the radius of *zone of attraction* while maintaining the same initial condition it results in decreasing the missing information. Increasing the *zone of attraction* causes the individuals to interact more with each other rather than being isolated and as a result the group tends to show more collective arrangment. This means there is more structural order and less missing information about the group formation. Similarly, fixing the radius of *zone of repulsion* and *zone of attraction* and increasing the radius of *zone of orientation* contributes to a reduction in the missing information (red line in Fig. 4b). This means that the expansion of orientation zone makes the individuals to align with each other and move parallel, which implies an increasing degree of internal order within the group. Consequently, the missing information decreases.

### From energy landscape to estimation of missing information, self-organization and complexity of three different natural groups

#### Bacteria

**Figure 5:**
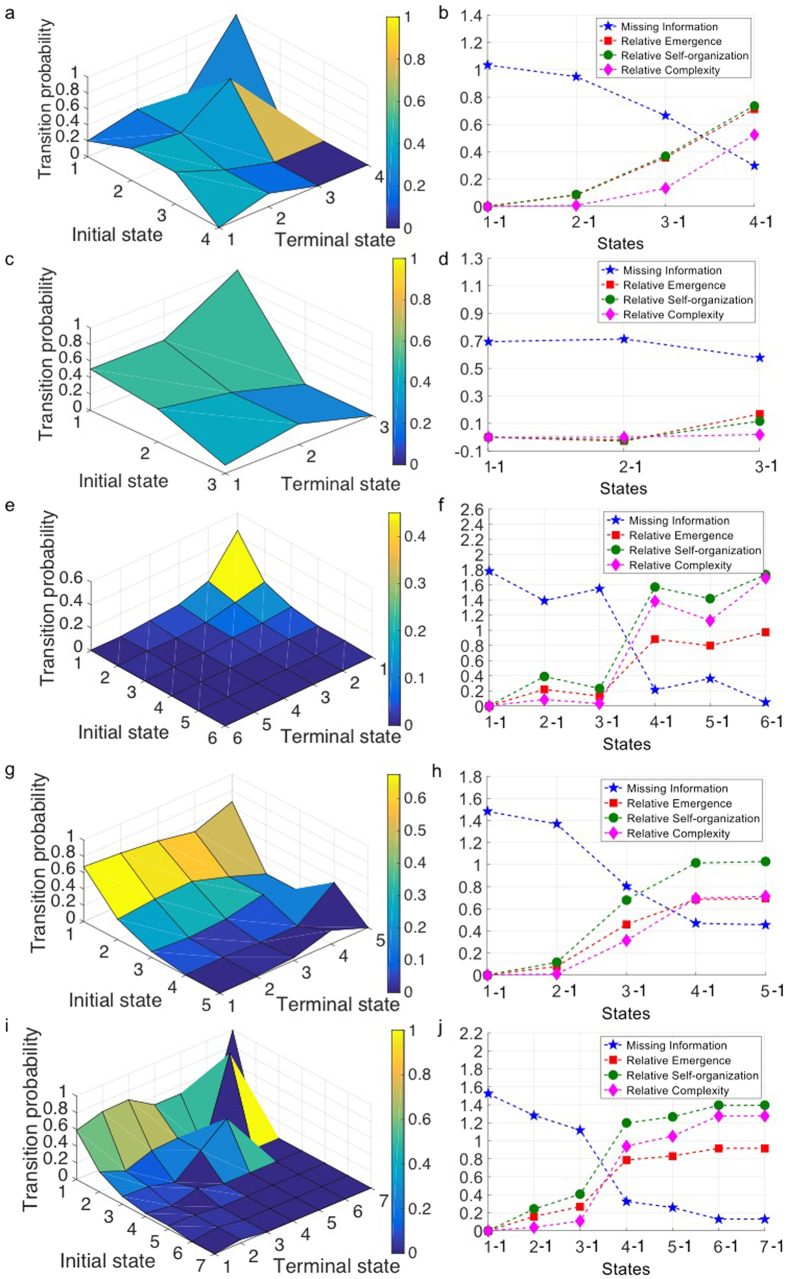
Transition probability matrix and complexity analysis for different natural collective motions. (**a**) Transition probabilities among the possible states for a collective group of 9 bacteria selected from a population density of 10^8^ bacteria/cm^3^ moving in an environment without chemoattractant gradient. (**b**) Complexity analysis for different states compared to the first identified state with lowest energy level for a group of 9 bacteria selected from a population density of 10^8^ bacteria/cm^3^ moving in an environment without chemoattractant gradient. This plot shows the level of change in missing information when the collective motion leaves each identified state to evolve to a new state (Note S1 in Supplementary Documents explains this in more details). It also demonstrates the relative emergence and relative self-organization and relative complexity of the swarm when evolving from any of the identified state to the first and most probable state. (**c**) Transition probabilities between the possible states for a group of 9 bacteria selected from a population density of 10^8^ bacteria/cm^3^ moving in an environment with chemoattractant gradient. (**d**) Complexity analysis for different states compared to the first identified state with lowest energy level for a group of 9 bacteria selected from a population density of 10^8^ bacteria/cm^3^ moving in an environment with chemoattractant gradient. (**e**) Transition probabilities between the possible states for a group of 9 pigeons in free flight. (**f**) Complexity analysis for different states compared to the first identified state with lowest energy level for a group of 9 pigeons in free flight. (**g**) Transition probabilities between the possible states for a group of 8 pigeons in home flight. (**h**) Complexity analysis for different states compared to the first identified state with lowest energy level for a group of 8 pigeons in home flight. (**i**) Transition probabilities between the possible states for a group of ants. (**j**) Complexity analysis for different states compared to the first identified state with lowest energy level for a group of ants.

Cellular collective groups can create complex patterns based on their simple behavioral rules. Different ways of communication and information transfer between them affects their individual behaviors and motion. To investigate and quantify the complexity of cellular groups, we study motion of *S. marcescens* bacteria with density of 10^8^ (bacteria/cm^3^) moving *in vitro*. We consider the motion of 9 bacteria (our analysis can be extended to a higher number if we are able to track and extract long time trajectories of all the individuals) in the experiments and consider it as a collective group for our analysis (see S. marcescens dataset from Methods for details). Next, we identify the possible states for the spatial formation of the selected group by analyzing the time trajectories of individuals’ motion. We investigate two different cases with and without distribution of chemoattractant in the environment. Considering the case without chemoattractant distribution in the environment (Fig. 5a), the analysis identifies four states for the formation of the group motion. When the transition probability of one state has higher level, it indicates that the amount of energy needed for the group of bacteria to create that formation is relatively lower. Based on our analysis, the first state has the lowest energy in the landscape compared to others. This state with the lower energy formation has the highest transition probability in the transition matrix, which means it is the most probable state for the group formation over time. We can order other states relatively to this state based on energy landscape from lowest energy to the highest. We categorize them into two groups of stable and transitioning states. When the group has higher probability to stay in the same state over time, we consider it as a stable state, which can be recognized as local equilibrium state for the group formation. These stable states have lower energy level compared to others. On the other hand, if it is not probable for the group to stay in the same state, we consider that as transient state. Because of the difference in the energy level, the group prefers to stay for a relatively longer time in stable states compared to transition states. Meanwhile, the group may shape the transition states while evolving between two different stable states.

Figure 5a and 5c demonstrate that adding chemoattractant to the environment, decreases the number of possible states for the group dynamics and the free energy landscape is more smooth compared to the case without chemoattractant. The existence of chemoattractant in the environment contributes to the preferable alignment of the bacteria motion with each other and causes the group to get more organized with less oscillatory and scattered motions.

Based on our analysis, we observe that the missing information of the first local equilibrium state is the lowest (Fig. 5b and 5d). Accordingly, for the transient state the missing information is higher compared to the stable states (see missing information section in Methods). Figure 5 shows the level of change in missing information when the collective group leaves any of the identified states to evolve to a new state (Note S1 in Supplementary Documents explains this in more details).

We define emergence for a collective group as being proportional to the structural order gained by the system and quantified in statistical terms with respect to ambiguity of the initial state^49^. Based on this definition, changing the system dynamics from one state to another can be interpreted as a way to transform or propagate the information. Figure 5b and 5d show the relative emergence of all possible states with respect to the most probable state, which has the lowest amount of missing information (see emergence section in Methods). Based on our analysis, high emergence means more dependencies in the group as a result of stronger interactions. This indicates that the group has more interdependent components in stable states with lower level of missing information compared to transition states. On the other hand, low emergence shows the group has more independent components, which represents the group in transition states.

We define a group to be self-organized when the internal dynamics of the group increases its organization over time. This could be a measure for collective group intelligence and we plan to investigate further in our future work. Our proposed free energy landscape analysis shows that the group is naturally attracted to be in stable states with lower energy compared to transitioning states with higher energy level. By considering these attractor stable states as organized states, the group will be self-organized through time (see self-organization section in Methods). Figure 5b and 5d show the level of increase in self-organization of the swarm going from any possible state to the first stable state with lower energy level (i.e., each point shows the amount of increase in group self-organization evolving from the corresponding state to the first stable one). The figure shows that the group gets more self-organized when it evolves from any possible state to the first and most probable one with the lowest level of energy. This shows that the bacteria group gets more self-organized over time.

The final purpose of our analysis is studying the complexity of a group motion. To quantify the degree of complexity for a group with specific types and possibly unknown or impossible to detect agent-to-agent interactions, we compute a complexity metric as the product between emergence and self-organization (see complexity section in Methods). Figure 5b and 5d show the relative complexity of all the possible states with respect to the first and the most stable state. Each point in this plot shows how complexity changes by evolving from the corresponding state (i.e., the states represented by that point) to the first stable one. This figure shows that the complexity metric exhibits an increasing tendency when the group evolves from transition states to stable ones. This shows that over time, the group tends to stay more in the stable states with higher complexity compared to other ones.

#### Flying Pigeon

Next, we analyze two different types of flying pigeon groups: free flight and home flight (see the Pigeon dataset from Methods for details). In free flight case, the pigeons are flying freely in the sky while in the home flight they are migrating from one region to another region. Figure 5e–h show that for free flight we have more dominant states compared to the home flight. This demonstrates that when the group has a destination and its goal is to reach its destination rather than just flying freely in the sky, it oscillates between less number of dominant spatial state formations.

Our analysis of the proposed information metric (see the results in Fig. 5f and 5h) demonstrates that the stable states have a lower degree of missing information and higher degree of emergence, self-organization and complexity compared to transition states. This means that over time, the group of pigeons, independent of their flight type, tends to have spatial formation/structure related to stable states which has lower energy, higher degree of complexity compared to the transition states.

#### Ant

Insect’s societies can be considered as an example of complex systems. For instance, a group of ants exhibit emergent characteristic at a higher level compared to the sum of emergent corresponding to all individuals separately. This means the group reacts like a single coherent entity in different situations (e.g., presence of attack to different part of the group)^50^. Therefore, scientists consider a group of ants as a single super-organism^51^. The individual ants in a group tend to form spatial organized structure (i.e. spatio-temporal states) with respect to each other. Using our framework, we can identify these spatio-temporal states, build their energy landscape and quantify their complexity. Regarding this, we analyzed a group of eight ants with identical role inside their population with our algorithm (see Ant dataset from Methods for details). Figure 5i shows the transition probability matrix. In this figure, the high peak points correspond to the lower energy levels in the landscape, meaning that the transition of the group among these states consumes less energy. Figure 5j shows the missing information and complexity analysis. We can see the same pattern meaning that the stable states have lower missing information and higher emergence, self-organization and complexity compared to transition ones.

It has been shown that the spatial organization of ants with different duties in a group strongly depends on their role^50^. Consequently, depending on the roles they play in the group they may form different structural states. One of the potential applications of our framework in the future can be studying the performance of ants with the same role inside their colony under different environmental conditions (e.g., attack to different parts of the group, migrating to new nest). We can also quantify the information transfer among members of different species inside a colony and compare the dependency of communication between them based on the role they are playing inside the group. These two potential applications of our framework remains for the future work due to lack of access to required data for these analyses.

## Discussion

Animals moving in a group are influenced by their social context meaning they adjust their motion in response to interactions with their neighbors and environment^52^. They keep a minimum distance from each other to avoid collision. Meanwhile, they have a long range attraction to others, which keeps them united as a group and prevent their isolation from the rest of the group. At the same time, they tend to align the direction of their motion with the ones near them to move in a synchronous fashion. These interactions between agents in the group are due to their sensory systems including vision, smell detection/chemical processing and sound. These multi-modal heterogeneous interactions among the agents cause the motion of the group to evolve through various spatio-temporal structures while moving as a synchronized and coherent entity without having a centralized controller.

Such synchrony and structural patterns in the group helps the individuals to amplify their sensitivity and reactions/agility to the environmental conditions while they have limited individual sensing and processing capabilities. This proves critical for their survival^52^,^53^. As an example, when a predator attacks the group, a small portion of the group senses the attack prior to the rest of the group. The efficiency of achieving a high degree of collective behavior helps to adapt faster to perturbation and decreases the reaction time of the whole group to the dangerous situations. In this case, they transform to a specific structural pattern to align more strongly with each other helping them to escape faster from the threat. As another example, such synchrony between them helps the group to identify the resources in the environment more efficiently. Hence, the spatial structuring within groups has important and evolutionary consequences^31^,^46^. From this perspective, it is crucial to be able to study the whole group considering their structure and to develop a mathematical framework for identifying and quantifying the information flow within the group.

Our mathematical framework helps to analyze various types of collective behavior exhibited by a group and identify/extract the possible spatio-temporal states that correspond to the highly interdependent group dynamics. Estimating the transition probability matrix between different states helps us to construct the energy landscape of the collective group evolving among these possible states over time. Relaying on this probabilistic characterization of the interdependency structure among various states, we quantify the missing information corresponding to the structural formation of a particular collective behavior for the first time. We show that when local interactions among individuals increase in strength, the individuals tend to align more with their neighbors and as a result the swarm gains more internal order. Therefore, the group structure does not change too much through time and as a result the number of possible states decreases and the missing information of the group structure decreases as well. We believe that this will help us understand how group of moving agents overcome the information bottleneck and plan to design new real experiments^54^.

We also quantify the missing information, emergence, self-organization and complexity of the group corresponding to each of its possible structural states. We show that over time the group tends to stay in stable states with lower level of energy; this corresponds to higher degree of self-organization and complexity compared to other possible states. Our analysis demonstrates that the complexity of the group formation increases over time, which could be attributed to the fact that the interactions are evolving or adapting to external cues. Our mathematical framework can help us understand the evolution of behavior of various complex systems, from human microbiome to road traffic and potentially also economic and social networks. An important applicability domain of the proposed framework is represented by the need for a robust mathematical formalism for quantifying the efficiency, adaptivity, robustness and agility of a swarm of artificial learning cells and comparing how two artificial groups with different heterogeneous interactions and learning capabilities can perform on different environments with various degrees of uncertainty.

Our framework could also serve as an initial step towards a solution to one of the main challenges in collective motion optimization and control. Communication between agents enables a decentralized control strategy for collective motion optimization. This causes the group of agents to self-organize and creates spatio-temporal patterns and ordered structures while following a good path at a specific time for their motion. This optimization observed in the group motion is a sign of intelligent behavior. According to Gerardo Beni^53^ an intelligent group can be considered as a large parallel computational system, which performs computation and motion in parallel. Computation and simulation time for an agent based model, which predicts the group performance from its initial state scales with the number of agents. If the number of agents is large enough then the computation time increases exponentially and also the possible outcome after a certain finite number of steps of evolution of the group is a NP-complete problem^32^,^42^,^55^,^56^. Therefore, control of such group with decentralized controllers is still a fundamental challenge, because there is often no obvious relation between the individual’s behavior and the final behavior of the whole group^32^. Our algorithmic strategy can be integrated into an engineering framework to be used to set the parameters that governs the dynamic of one agent and its corresponding interactions with other agents to achieve a certain degree of emergence, self-organization and complexity. For instance, one could combine an agent based model^22^,^31^ (where the characteristics and behavior of each individual are driven by several knobs) and macroscopic analytical model^42^,^57^ to describe the collaboration in a group of reactive robots. In the next step, use our framework and quantify the emergence, self-organization and complexity as a function of the control knobs and size of the group. This combined analysis can enable the identification of the critical design considerations. Consequently, by playing with the local interactions between the agents, we can regulate the system to evolve towards desired states and control the corresponding free energy landscape. Controlling over the energy landscape implies following a few rules of interaction that contribute to a particular set of states with desired degree of self-organization and complexity. This remains for future work.

Using our framework can help to build, characterize and optimize a group of robots with much simpler components with a decentralized control characteristic and replace the complex centralized control systems to perform the same task. By comparison, simplicity of the agents and decentralized control characteristics of the group make it possible for the group to adapt dynamically better to the environment and recover from different disturbances in the environment^53^. Therefore, the collective group systems can be more reliable to survive through disturbance compared to centralized control systems.

## Methods

### Simulation

**Figure 6:**
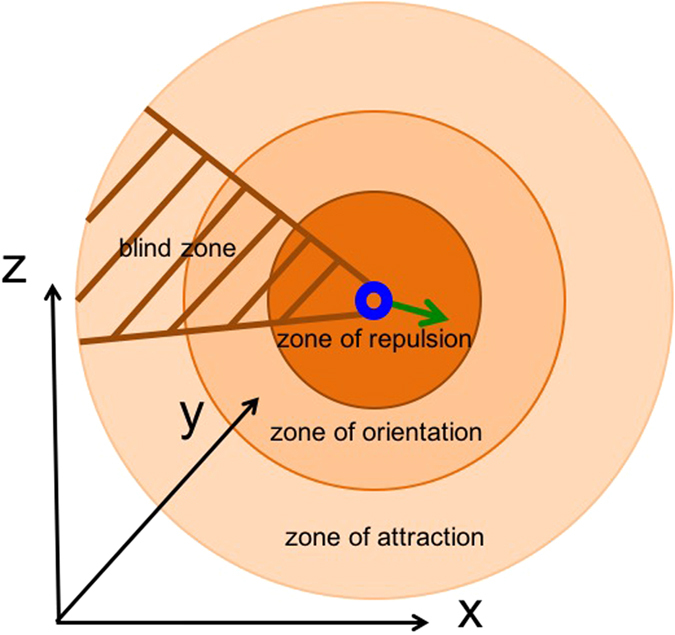
Different zones of interaction around each individual in a group of agents moving in three-dimensional space in a model proposed by Couzin and his coworkers^31^: *Zone of repulsion*, *zone of orientation* and *zone of attraction*. There is a blind zone behind each individual, in which they do not sense and react towards other neighbors in that area.

The simulations are based on a well-known agent-based model proposed by Couzin and his coworkers^31^. The details of the model are as follow. Consider *N* individuals in a group (*i* = 1, 2, 3,…, *N*) with position vector *p*_*i*_(*t*) and direction vector *d*_*i*_(*t*) at each time. The desired direction of each individual for the next time step based on local interaction between them is *w*_*i*_(t + *τ*) with *τ* representing the time step. This model considers three different zones around each individual (Fig. 6). The first spherical zone called *zone of repulsion* and the individual is located at the center of it. If there are other neighbors in the *zone of repulsion* of an individual, the individual moves away from them to keep a minimum distance and prevent collision. The second spherical zone is the *zone of orientation*. If there is no other neighbor in the *zone of repulsion* of an individual, then the individual tries to align itself with other neighbors in its *zone of orientation*. The third spherical zone is the *zone of attraction*. The attraction between an individual and its neighbors in this zone results in the coherence of the group. Considering these three regions, there is a blind volume behind the individual in which the individual does not sense and respond to other neighbors in this zone.

In this model, variables *n*_*r*_, *n*_*o*_ and *n*_*a*_ represent correspondingly the number of neighbors in zone of repulsion, orientation and attraction of the agent. Variable *w*_*r*_(*t* + *τ*) represents the desired direction of individual *i* with respect to repulsion from others in repulsion zone.


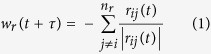



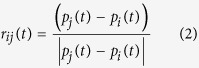


When there are some neighbors in *zone of repulsion (n*_*r*_ ≠ 0), the individual *i* only reacts with respect to them. As a result, the desired direction *w*_*i*_(*t* + τ) = *w*_*r*_(*t* + τ) can be quantified from equation (1) and equation (2). If there is no individual in the *zone of repulsion*, then the desired direction will be defined based on neighbors in zone of orientation and attraction (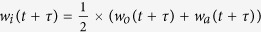). *w*_*o*_(*t* + τ) and *w*_*a*_(*t* + τ) can be quantified from equation (3) and equation (4).


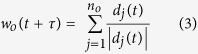



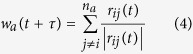


Considering the desired direction vector at each time step, if *w*_*i*_(*t* + *τ*) is less than maximum turning rate *θ*, then *d*_*i*_(*t* + *τ*) = *w*_*i*_(*t* + τ). On the other hand, if desired direction vector exceeds the maximum rate, then the individual rotates by angle of τ×θ towards the desired direction.

### Free energy landscape

Our framework generalizes the method presented by Akinori Baba and coworkers^13^,^47^ and constructed the strategy to estimate the free energy landscape for a group of *N* agents moving in a three-dimensional space. In the following, we provide a brief overview of the procedure we used to identify and extract the states from time series of agents in the group. First, we divide the time series containing the location of all the agents denoted by *r*(*t*) to sub-intervals centered at time *t*_*c*_ with time window [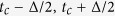], where Δ is the preferential time scale (Fig. 1a). In the next step, we construct the probability density function of the location of all the agents in the group corresponding to each sub-interval (i.e. *p*_*i*_) and based on that we find cumulative distribution function (CDF) of the agents’ location in the space. We also estimate the CDF corresponding to the position for the entire group through the whole time in the same way. Based on Kantrovitch distance *d*_*K*_ we compare the CDF of sub-intervals with whole time series CDF and cluster the sub-intervals based on the similarities (equation (5))^58^.


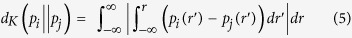


We consider each of the clusters as a spatio-temporal state for the group dynamics (Fig. 1b). We calculate the escape time of each state, meaning the time between when the system enters and leaves each cluster.

We calculate the residential probability *P*_*i*_ of the *i*th state and transition probabilities *P*_*ij*_ from the *i*th state to the *j*th state (Fig. 1c). Based on these probabilities, we estimate the free energy landscape by quantifying the energy level in each state (*F*_*i*_) from equation (6) and energy barrier for the group while evolving from state *i* to state *j (F*_*ij*_) from equation (7) 47.


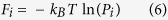



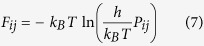


In equation (6) and (7), symbol *k*_*B*_ represents Boltzman constant. Symbols *h* and *T* are Plank constant and temperature, respectively. Based on these energy levels we can estimate the free energy landscape of the group evolving between different states.

### Missing Information

In general, missing information can be defined as quantifiable structure or pattern in a system^38^,^48^,^59^. It can be used as a measure of internal order of a system and uncertainty. According to Shannon, missing information can be defined from equation (8).


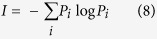


We define missing information for a collective motion as the level of missing communicated information between the agents due to their short-range and long-range interactions. This can be interpreted as the amount of information needed to specify the coupling between the agents and as a result the exact structural state of the group forming a specific spatial arrangement.

In our framework, we find the dominant states for a collective motion dynamics from energy landscape analysis. Based on the landscape, we quantify: (1) missing information of each state (2) missing information of the entire motion of group combining all the possible states. To compute the missing information for each state we find the probability transition matrix *P* of the swarm evolving from one state to the others. Then we find the missing information related to each state *i* (I_*i*_) from this probability transition matrix using equation (9):


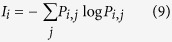


Next, we find the missing information related to the entire group motion considering all the possible states. We define matrix Q containing the probability of all the possible cases of transitioning from one state to the other, independent of the initial state in the transition. The missing information for entire group motion (*I*) can be quantified by equation (10):


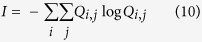


It is important to emphasize the difference between matrix *P* from *Q*. In probability transition matrix *P* the sum of all the elements in each row is equal to one, while in probability matrix *Q* the sum of all the elements in the entire matrix is equal to one. The difference is due to the way of normalizing the probability matrix.

### Emergence

Emergence in a system generally refers to some information or characteristics of a system that appear in some states while they are not present in other states of the system^48^,^49^,^60^. Emergence of a system can be quantified by equation (11).


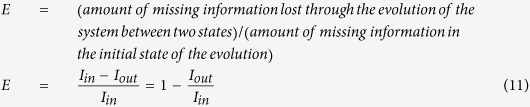


For a collective motion, we define emergence as being proportional to the structural order gained by the system and quantified in statistical terms with respect to the ambiguity of the initial state. Therefore, higher emergence shows more interdependence in the group due to stronger interactions between the agents. This shows the transferred information between the agents results in the dependencies of their motion in the group. In our framework we find the relative emergence in each identified state with respect to first state with the highest probability and lowest level of energy in the landscape.

### Self-organization

We call a system self-organize when the internal dynamics of a system increases its organization in time^48^. Equation (12) quantifies the self-organization of a system^48^.


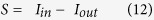


For a collective motion, we consider self-organization as a measure for the transformed information between the agents into the internal order of the group structure. This could be considered as a measure for group intelligence. In our framework we find the level of increase in self-organization of the group going from any possible state to the first and most probable state to be able to compare the self-organization of different states in a collective motion.

### Complexity

In general, Complexity represents the balance between emergence (presents variety) and self-organization (presents order) of a system^48^,^59^,^61^,^62^. In other words, it is a balance between ordered and chaotic dynamics. Complexity of a system can be quantified by equation (13)^48^.


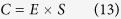


We consider the complexity for a collective motion as a combination of interdependency between the agents and internal order in the group structure as a result of transferred information between the agents due to short-range and long-range interactions. In our framework, we quantify the relative complexity of each state in a collective motion with respect to the complexity of the first possible state from emergence and self-organization corresponding to that state.

### *S. marcescens* dataset

We obtain the data for a group of *S. marcescens* moving in three-dimensional space from Edwards *et al*.^63^ and Zhuang *et al*.^64^.

### Pigeon dataset

We obtain the data for group of Pigeons Flying in three-dimensional space from Nagy *et al*.^65^.

### Ant dataset

We obtain the data for ant trajectory from Queen Mary University database directory from the following link^66^: ftp://motinas.elec.qmul.ac.uk/pub/mtt_results/ant_tracking_res.zip\

## Additional Information

**How to cite this article**: Koorehdavoudi, H. and Bogdan, P. A Statistical Physics Characterization of the Complex Systems Dynamics: Quantifying Complexity from Spatio-Temporal Interactions. *Sci. Rep.* **6**, 27602; doi: 10.1038/srep27602 (2016).

## Supplementary Material

Supplementary Information
