# Supplementary material for: A Statistical Physics Characterization of the Complex Systems Dynamics: Quantifying Complexity from Spatio-Temporal Interactions
Source: Sci Rep. 2016 Jun 14;6:27602. doi: 10.1038/srep27602 (PMC4906350; doi:10.1038/srep27602)
Supplement: Supplementary Information [file srep27602-s1.pdf]

Supplementary Documents

A Statistical Physics Characterization of the Complex Systems Dynamics: Quantifying Complexity from Spatio-Temporal Interactions

Hana Koorehdavoudi[1], Paul Bogdan[2,*]

[1]Department of Mechanical Engineering, University of Southern California, Los Angeles, CA 90089-2560, USA.

[2]Department of Electrical Engineering, University of Southern California, Los Angeles, CA 90089-2560, USA, (pbogdan@usc.edu)

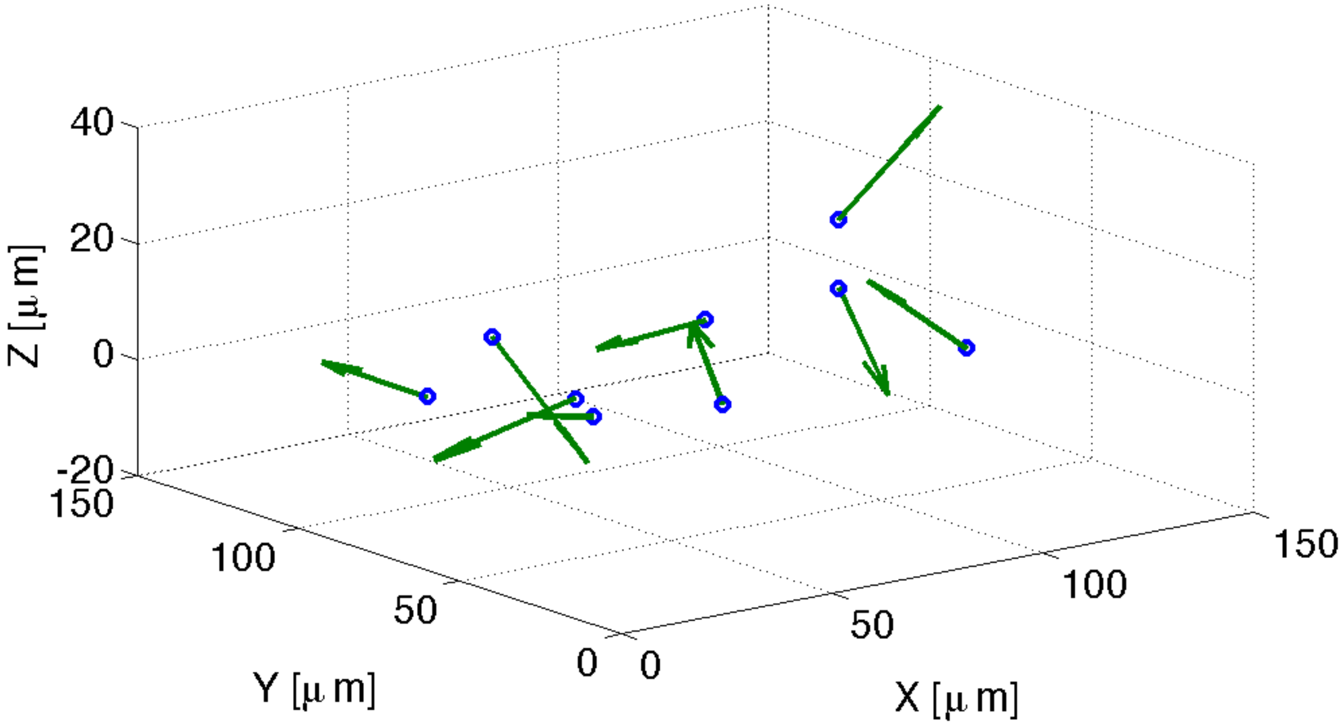

Figure S1. Collective pattern of a group of bacteria moving on a three-dimensional space. The position and direction of motion for a group of 9 bacteria extracted from a population density of 10^8 bacteria/cm^3 swimming in an environment without chemoattractant gradient.

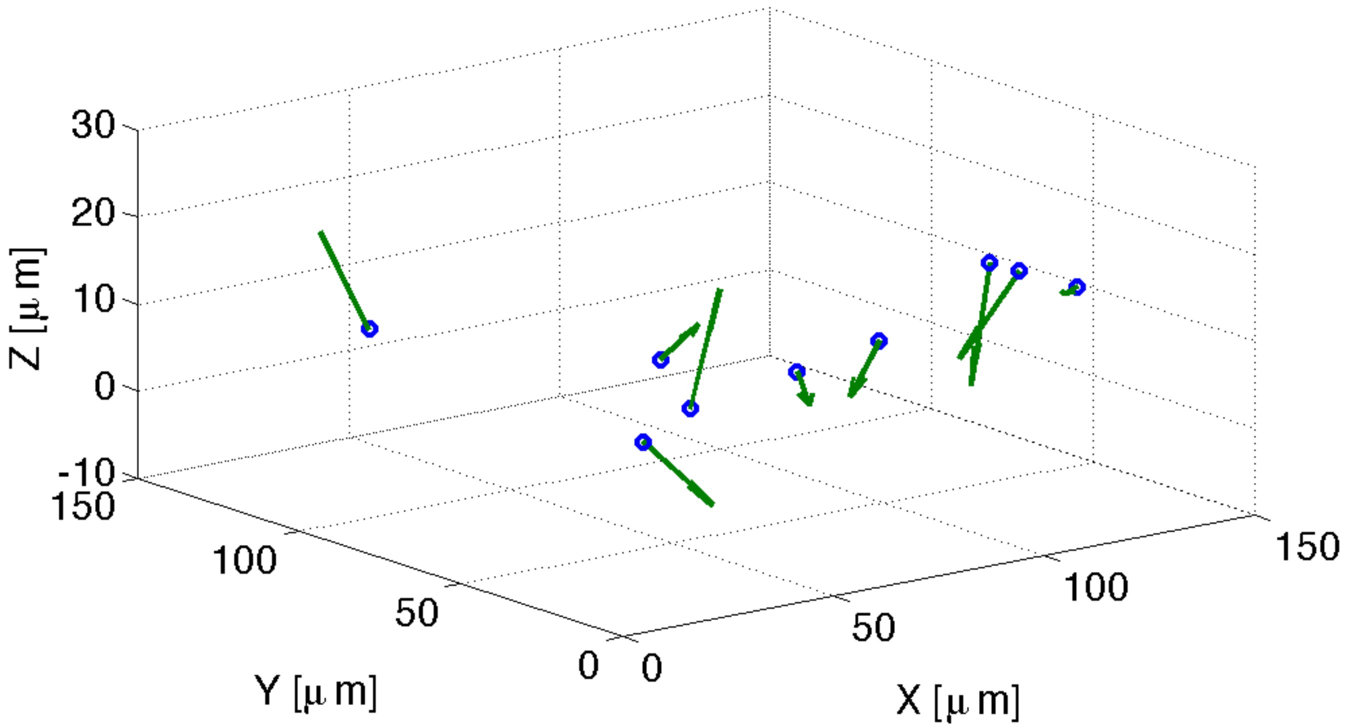

Figure S2. Collective pattern of a group of bacteria moving on a three-dimensional space. The position and direction of motion for a group of 9 bacteria extracted from a population density of 10^8 bacteria/cm^3 swimming in an environment with chemoattractant gradient.

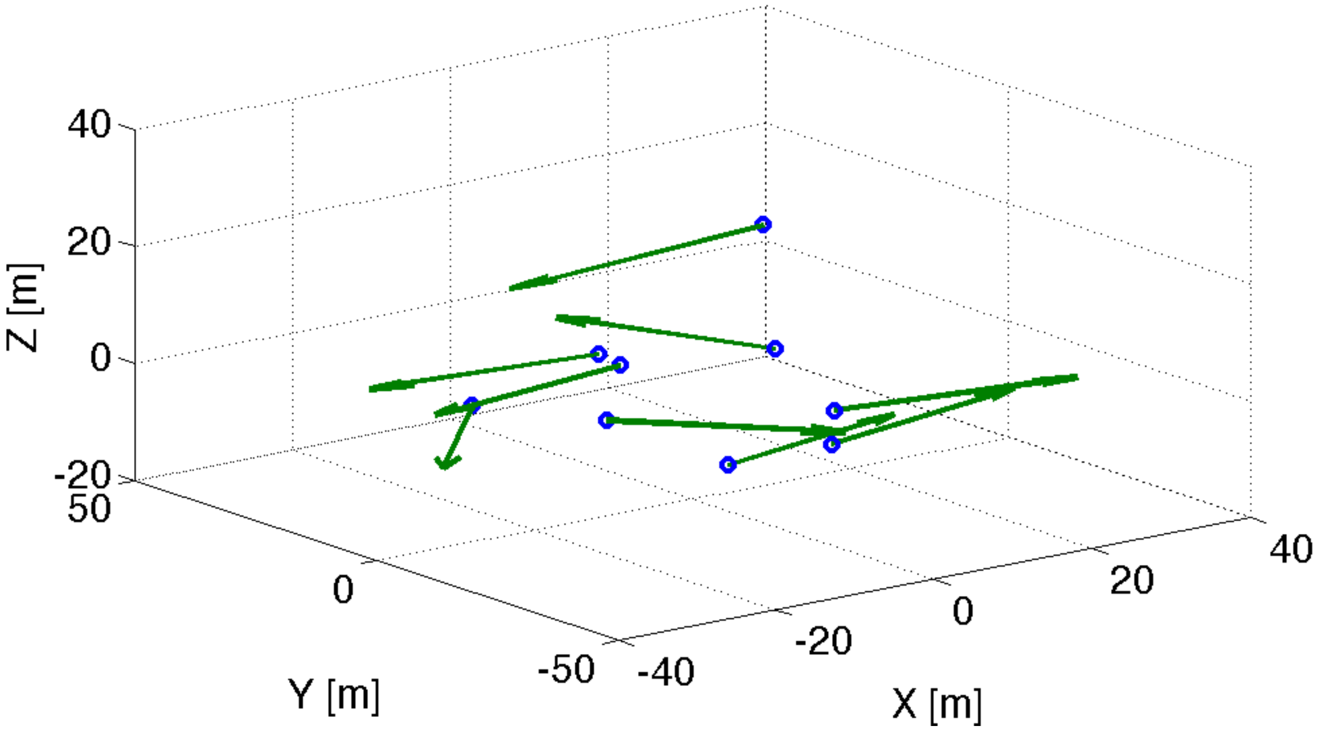

Figure S3. Collective pattern of a group of pigeons in free flight. The position and direction of motion with respect to center of the group for a group of 9 pigeons in free flight.

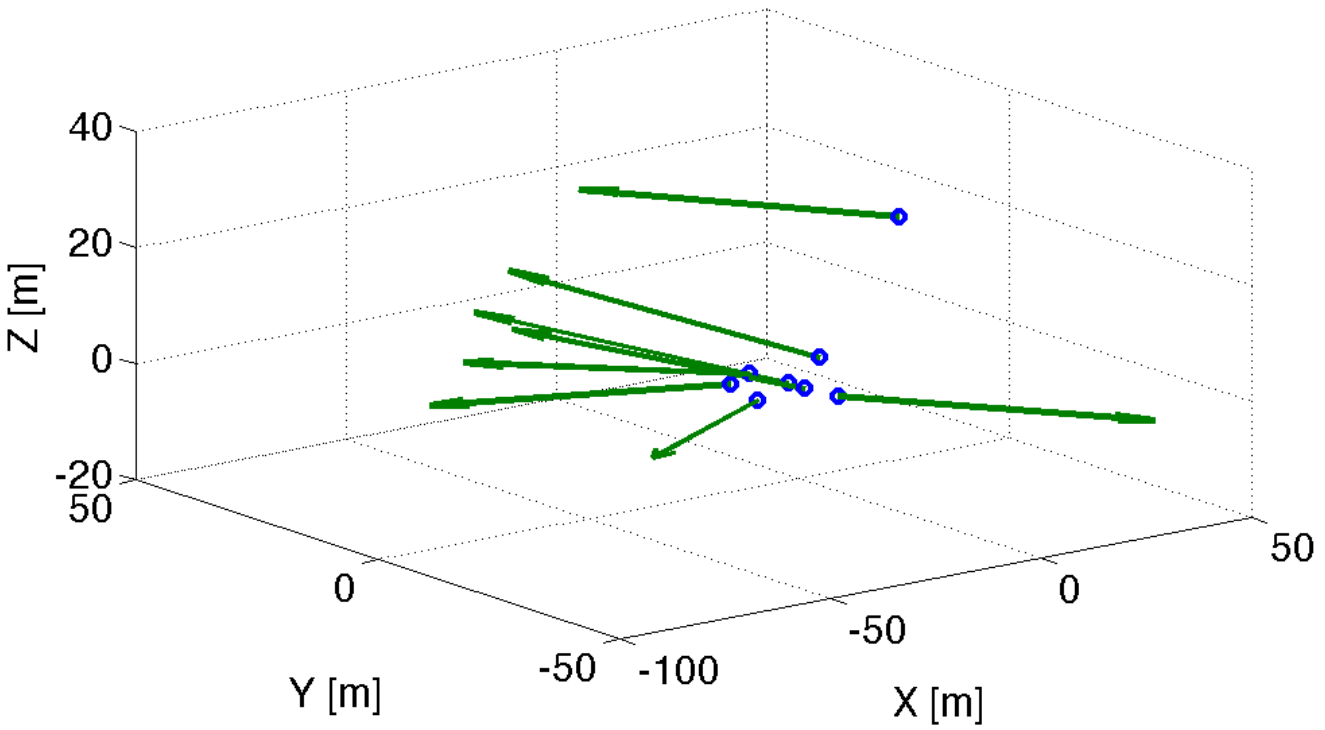

Figure S4. Collective pattern of a group of pigeons in home flight. The position and direction of motion with respect to center of the group for a group of 8 pigeons in home flight.

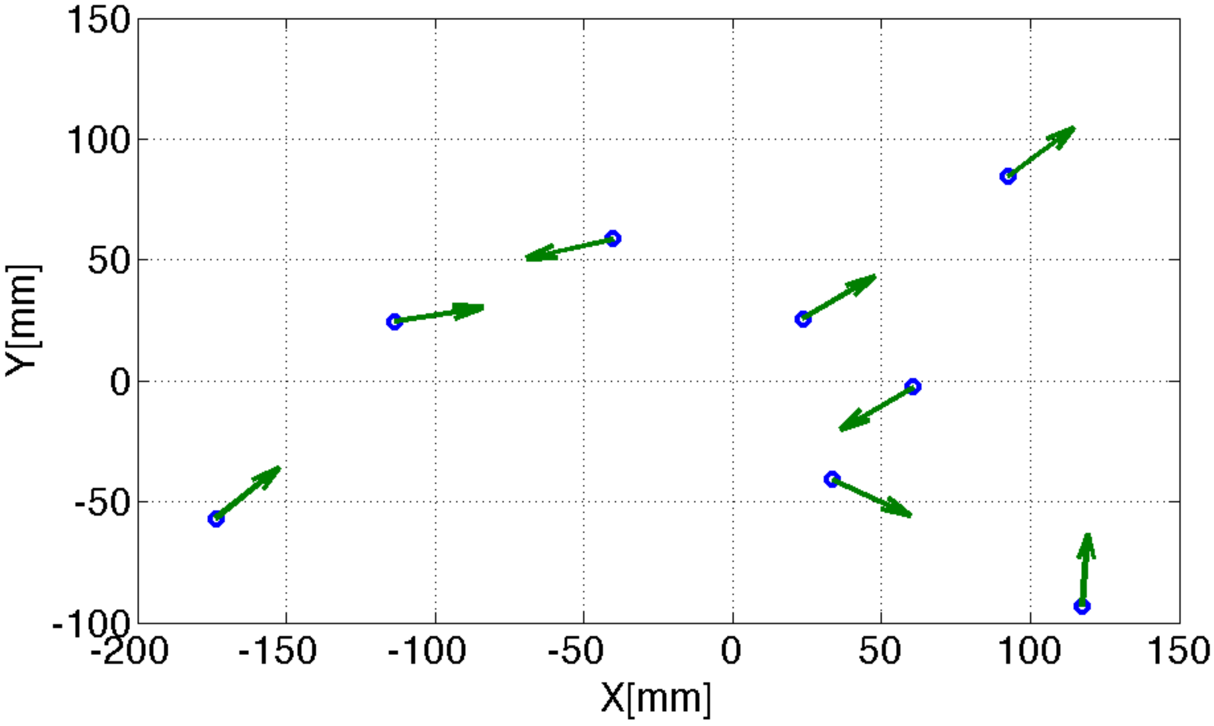

Figure S5. Collective pattern of a group of ants moving on a two-dimensional surface. The position and direction of motion for a group of 8 ants moving on a surface in a time step.

Note S1. Level of change in missing information

Figure S6 makes the concept of **level of change in missing information** when the group motion **leaving state i** to any possible state more clear. In this figure, when the dynamic of the group leave state 1 and evolve to any other possible state, the level of missing information increases on average, irrespective of the terminal state. Therefore, we can conclude that the absolute missing information of state 1 compared to other states is lower.

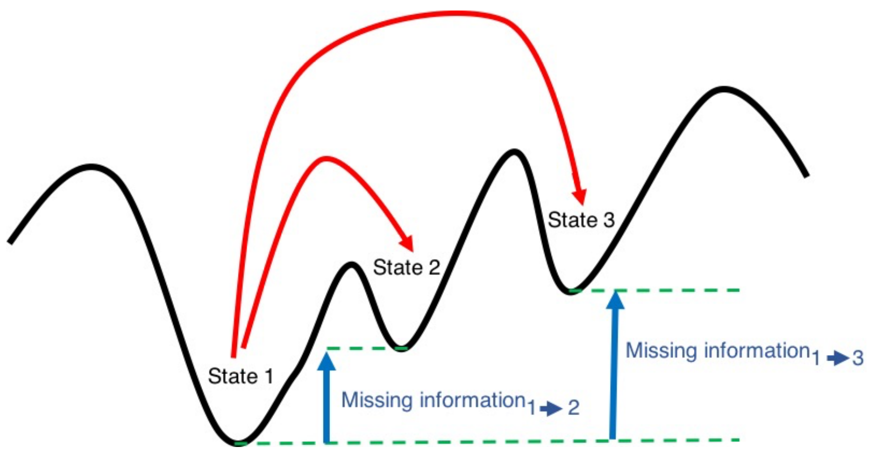

Figure S6. Level of change in missing information through the evolution of the dynamic of collective group from state 1 to other states.
